# Macrophage to myofibroblast transition contributes to subretinal fibrosis secondary to neovascular age-related macular degeneration

**DOI:** 10.1186/s12974-020-02033-7

**Published:** 2020-11-25

**Authors:** Karis Little, Maria Llorián-Salvador, Miao Tang, Xuan Du, Stephen Marry, Mei Chen, Heping Xu

**Affiliations:** The Wellcome-Wolfson Institute for Experimental Medicine, School of Medicine, Dentistry & Biomedical Sciences, Queen’s University Belfast, 97 Lisburn Road, Belfast, BT9 7BL UK

**Keywords:** Age-related macular degeneration, Retinal fibrosis, Macrophage to myofibroblast transition, Inflammation, Complement

## Abstract

**Background:**

Macular fibrosis causes irreparable vision loss in neovascular age-related macular degeneration (nAMD) even with anti-vascular endothelial growth factor (VEGF) therapy. Inflammation is known to play an important role in macular fibrosis although the underlying mechanism remains poorly defined. The aim of this study was to understand how infiltrating macrophages and complement proteins may contribute to macular fibrosis.

**Methods:**

Subretinal fibrosis was induced in C57BL/6J mice using the two-stage laser protocol developed by our group. The eyes were collected at 10, 20, 30 and 40 days after the second laser and processed for immunohistochemistry for infiltrating macrophages (F4/80 and Iba-1), complement components (C3a and C3aR) and fibrovascular lesions (collagen-1, Isolectin B4 and α-SMA). Human retinal sections with macular fibrosis were also used in the study. Bone marrow-derived macrophages (BMDMs) from C57BL/6J mice were treated with recombinant C3a, C5a or TGF-β for 48 and 96 h. qPCR, Western blot and immunohistochemistry were used to examine the expression of myofibroblast markers. The involvement of C3a-C3aR pathway in macrophage to myofibroblast transition (MMT) and subretinal fibrosis was further investigated using a C3aR antagonist (C3aRA) and a C3a blocking antibody in vitro and in vivo.

**Results:**

Approximately 20~30% of F4/80^+^ (or Iba-1^+^) infiltrating macrophages co-expressed α-SMA in subretinal fibrotic lesions both in human nAMD eyes and in the mouse model. TGF-β and C3a, but not C5a treatment, significantly upregulated expression of α-SMA, fibronectin and collagen-1 in BMDMs. C3a-induced upregulation of α-SMA, fibronectin and collagen-1 in BMDMs was prevented by C3aRA treatment. In the two-stage laser model of induced subretinal fibrosis, treatment with C3a blocking antibody but not C3aRA significantly reduced vascular leakage and Isolectin B4^+^ lesions. The treatment did not significantly alter collagen-1^+^ fibrotic lesions.

**Conclusions:**

MMT plays a role in macular fibrosis secondary to nAMD. MMT can be induced by TGF-β and C3a but not C5a. Further research is required to fully understand the role of MMT in macular fibrosis.

**Graphical abstract:**

Macrophage to myofibroblast transition (MMT) contributes to subretinal fibrosis. Subretinal fibrosis lesions contain various cell types, including macrophages and myofibroblasts, and are fibrovascular. Myofibroblasts are key cells driving pathogenic fibrosis, and they do so by producing excessive amount of extracellular matrix proteins. We have found that infiltrating macrophages can transdifferentiate into myofibroblasts, a phenomenon termed macrophage to myofibroblast transition (MMT) in macular fibrosis. In addition to TGF-β1, C3a generated during complement activation in CNV can also induce MMT contributing to macular fibrosis. RPE = retinal pigment epithelium. BM = Bruch’s membrane. MMT = macrophage to myofibroblast transition. TGFB = transforming growth factor β. a-SMA = alpha smooth muscle actin. C3a = complement C3a. 
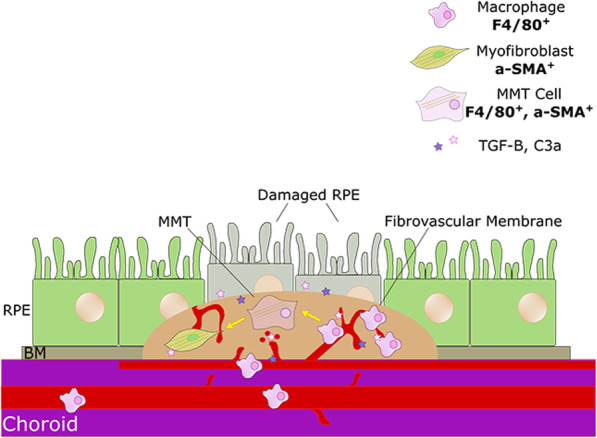

## Background

Age-related macular degeneration (AMD) is a disease which results in loss of central vision in the elderly. It is estimated that around 288 million people will be diagnosed with AMD worldwide by the year 2040 [1]. Around 10% of patients with AMD suffer from the neovascular form of the disease (nAMD), which is characterized by the growth of abnormal blood vessels in the macula and leaves patients more at risk of severe vision loss [2]. If nAMD remains untreated, eventually, patients will lose sight and develop macular fibrosis [3, 4]. The introduction of anti-vascular endothelial growth factor (anti-VEGF) therapy has revolutionized nAMD therapy [5, 6]. However, around one-third of patients still develop macular fibrosis even with anti-VEGF therapy [7]. Macular fibrosis remains to be a major clinical challenge in nAMD management. With an ageing population and predicted increases in AMD patients, this challenge is set to become an increasing problem.

Macular fibrosis lesions contain blood vessels and hence are known as fibrovascular lesions [8–10]. The specific cues involved in the transition of the diseased vessels to a fibrovascular scar remain unknown. As well as blood vessels, macular lesions contain infiltrating immune cells, myofibroblasts and excessive amounts of extracellular matrix (ECM) proteins such as collagens, fibronectin and laminin [11–13]. Myofibroblasts are the active form of fibroblasts, which do not exist in the macula. It has been hypothesized that myofibroblasts in macular fibrosis originate from differentiation of either resident retinal cells (e.g., retinal pigment epithelial cells) or infiltrating inflammatory cells [8, 14] although direct evidence is lacking.

A previous study has shown that 61% of human choroidal neovascularization (CNV) lesions contained macrophages [15]. Macrophages constitute 20% of all cells in experimental CNV, and ~ 70% of infiltrating macrophages originate from the bone marrow [16]. Macrophage is believed to play an important role in macular fibrosis development although the underlying mechanism remains to be determined. Recent studies have shown that macrophages can transdifferentiate into myofibroblast-like cells after TGF-β stimulation (macrophage to myofibroblast transition; MMT) and that this process contributes to kidney fibrosis [17–19].

Complement system dysregulation has been identified as a key inflammatory pathway in AMD [20–23]. Our group has shown that patients with macular fibrosis in nAMD have increased levels of complement components including 3a, 4a and 5a [24]. Neutralising C3a and C5a during experimental laser-induced CNV reduced neovascular lesions [25]. It has been reported that C3a can induce epithelial to mesenchymal transition (EMT) differentiation in proximal tubule epithelial cells (PTECs) through the C3a receptor—C3aR1 [26]. In this study, we investigated the role of MMT in macular fibrosis. We further examined the influence of C3a and C5a in MMT in bone marrow-derived macrophages and in a mouse model of subretinal fibrosis.

## Methods

### Animals

C57BL/6J mice (male and female) aged between 2 and 3 months were used for this study. All animals were housed and bred in the Biological Service Unit of Queen’s University Belfast and exposed to a 12-h light/dark cycle with free access to food and water. All procedures were conducted under the regulation of the United Kingdom (UK) Home Office Animals Scientific Research Act 1986 and in accordance with the ARVO statement for the Use of Animals in Ophthalmic and Vision Research.

### Two-stage laser model of subretinal fibrosis

A two-stage laser model was carried out as previously described [9]. Briefly, laser CNV was induced. The settings for the laser were as follows: laser power—250mv, duration—0.1 s, and spot size—100 μm. Three laser spots were delivered per eye. Seven days later, a second laser burn was applied to each CNV lesion, using the same laser configuration.

### Inhibition of C3a in an in vivo model of subretinal fibrosis

Subretinal fibrosis was induced using the two-stage laser model as described above. Immediately after the second laser injury (day 0), mice received an intravitreal injection of a C3a blocking agent using the method described previously [27, 28]. A second injection was performed 20 days later (day 20).

The study groups were as follows: (1) anti-mouse C3a (rat IgG2a, clone 3/11; HyCult Biotechnology, Uden, The Netherlands, 2 μg/μl/eye) [25]; (2) rat IgG control (low endotoxin Bio-Rad, USA, 2 μg/μl/eye); (3) C3aR antagonist (C3aRA) (SB290157—Trifluoroacetate Salt, Cayman Chemical, USA, Cat No: 15783, 1 μg/μl/eye); (4) 1% dimethyl sulfoxide (DMSO) control (1 μl/eye); and (5) no treatment control group. Doses used were as previously described [25].

Fundus images were collected on day 20 using Micron IV system and the Discover 2.2 Programme (Phoenix Technology Group, USA). Fundus fluorescein angiography (FFA) images were collected using the same system. FFA was carried out 5 min after intra-peritoneal injection of 100 μl of 10% sodium fluorescein (Sigma-Aldrich, Gillingham, UK, Cat No: F6377). Exposure level was also kept consistent between animals. ImageJ was used to measure the leakage from each lesion, in a masked fashion. Mean grey value per lesion was compared between traditional laser CNV and two-stage laser groups. The findings were confirmed by a blinded, independent researcher.

Eyes were collected at 30 days post the second laser and fixed in 2% paraformaldehyde (PFA) (Sigma-Aldrich, Cat No: 158127). The size of fibrotic lesion was measured using area measurements of collagen-1^+^ and Isolectin-B4^+^ lesion size on retinal pigment epithelium (RPE) flatmounts.

### Human samples

Human eye samples with nAMD were obtained from San Diego Eye Bank. This study was carried out within the parameters of the Declaration of Helsinki, and patient tissues were stored in accordance with the Human Tissue Act (2004). The research was approved by the Ethical Review Boards of Queen’s University Belfast. The eyes were maintained in formalin. Upon arrival, the eyes were dissected and embedded in paraffin wax. Tissue sections were stored at 4 °C in a fridge designated for human tissue samples until required for staining. The results in this paper are representative of a single human sample. Haematoxylin and eosin (H+E) staining was carried out using Sigma-Aldrich H3136 Haematoxylin and BDH/VWR 95057-848 Eosin. Masson’s trichrome staining was performed on cryosections and wax sections using the Abcam trichrome staining kit according to the manufacturer’s instructions (Abcam, Cambridge, UK, Cat No: ab150686).

### Immunohistochemistry

Antigen retrieval was carried out by boiling slides in antigen retrieval buffer (0.05% citraconic acid, pH 7.4) (Sigma-Aldrich, Cat No: C82604) for 30 min, or enzymatic antigen retrieval method was used for rat anti-mouse F4/80 using 20 μg/ml proteinase K (Thermo Fisher Scientific, Loughborough, UK, Cat No: 17916) in Tris-Edta (TE) buffer for 2 min.

Following antigen retrieval, retinal sections were blocked using 5% BSA (Sigma-Aldrich, Cat no: A3803-10G) for 1 h at room temperature. Samples were incubated in primary antibody (Table 1) overnight at 4 °C, followed by incubations with secondary antibody (Table 1) in the dark at room temperature for 1 h. The sections were mounted in Vectashield with 4′,6-diamidino-2-phenylindole (DAPI; Vector Laboratories, USA, Cat no: H-1200). RPE flatmounts were stained using our protocol, as previously described [29]. Lesion size measurement in RPE flatmount was carried out as previously described [9]. The measurement was confirmed by an independent, blinded researcher.

**Table 1:** Antibody conditions for tissue staining

Ab name	Species	Company	Secondary antibody	Company
Collagen-1	Rabbit	Abcam: ab34710	594 Donkey anti-rabbit or 488 donkey anti-rabbit	Jackson: 711-585-152 Jackson: 712-545-150
F4/80	Rat	Bio-Rad: MCA497RT	594 Donkey anti-rat IgG or 488 donkey anti-rat	Jackson: 712-585-150 Jackson: 712-545-150
α-SMA	Mouse	Sigma-Aldrich: C6198	-Cy3 conjugated	–
Iba-1	Rabbit	Wako: 013-2769	488 Donkey anti-rabbit	Jackson: 712-545-150
C3a	Rat	BD Biosciences: 558251	Goat anti-rat-594	Invitrogen: A11007
C3aR	Chicken	Peninsula Laboratories International: T-2301	Biotinylated anti-chicken followed by streptavidin 488	Jackson: 703-065-155 ThermoFisher: S11223
Biotinylated Isolectin-B4	–	VectorLabs: B-1205	Streptavidin-488	ThermoFisher: S11223
TGF-β	Rabbit	Abcam: ab92486	594 Donkey anti-rabbit	Jackson: 711-585-152

### Culture of bone marrow-derived macrophages (BMDMs)

The bone marrow was isolated from healthy 8–12-week donor C57BL/6J mouse using the protocol previously described by our group [30]. Briefly, the bone marrow was flushed from the femur and tibia. After removing red blood cells, the cells were cultured in DMEM (Cat No: 41965039) supplemented with 1% penicillin-streptomycin (Cat No: 15140122), 15% foetal calf serum (FCS; Cat No: 10270106) (all from Thermo Fisher Scientific) and 20% L929 supernatant. Seven days later, the phenotype of BMDMs was checked by flow cytometry (FACS BD CANTO II, BD Biosciences, San Jose, USA) using the following antibodies: F4/80 conjugated eFluor 450 (Thermo Fisher Scientific, Cat no: 48-4801-80) and CD11b conjugated PE-Cy7 (BD Biosciences, Cat no: 552850).

### C3a treatment and inhibition in vitro

BMDMs cultured in 10% FCS were treated with 10 ng/ml recombinant mouse C3a (Cat No: 8085-C3) or C5a (Cat No: 2150-C5-025/CF) for 48–96 h. Ten nanograms per milliliter of TGF-β1 (Cat No: 7666-MB-005) (all from R&D Systems, Abingdon, UK) was used as a positive control. C3aR antagonist (C3aRA) SB 290157 (trifluoroacetate salt) (Cayman Chemical, Michigan, USA, Cat No: 15783) was dissolved in DMSO according to the manufacturer’s instructions. A final concentration of 10 μM was added to BMDMs in culture. The corresponding percentage of DMSO was 0.005%. Cells were plated in 6-well plates for the specified time with or without treatments before being collected for qPCR or Western blotting, or in 24-well plates for immunocytochemistry.

### Real-time PCR

Total RNA was extracted from cells using the RNeasy plus mini kit (Qiagen, Netherlands, Cat No: 74134). cDNA was synthesized using the SuperScript™ II Reverse Transcriptase kit (Thermo Fisher Scientific, Cat No: 18064014). Quantitative PCR (qPCR) was carried out using Lightcycler® 480 probes master mix as per the manufacturer’s instructions (Roche, Basel, Switzerland, Cat No: 04707494001). Primer sequences are shown in Table 2. TGF-β gene expression was analysed using a TaqMan probe (Roche, Cat No: 317139).

**Table 2:** Gene sequences for qPCR

Gene	Forward sequence	Reverse sequence
GAPDH	TGGCAAAGTGGAGATTGTTGC	AAGATGGTGATGGGCTTCCCG
α-SMA	TGGCACCACTCTTTCTATAACG	GGTCATTTTCTCCCGGTTGG
Fibronectin	CCACTTCCCCTTCCTGTACA	ATCGTAGTTCTGGGTGGTGC
Collagen-1	GTGGCGGTTATGACTTCAGC	GGCTGCGGATGTTCTCAATC
C3	ACCTTACCTCGGCAAGTTTCT	CTGCTGGTCAGGCTCCTC

### Immunohistochemistry staining

BMDMs were fixed for 20 min in 2% PFA at room temperature. After washing, the cells were blocked in 1% BSA (Sigma-Aldrich, Cat no: A3803-10G) diluted in PBS with 0.02% Triton-x for 1 h at room temperature, followed by 1-h room temperature incubation with primary antibody (Table 1). Secondary antibody (Table 1) was applied for 1 h room temperature after thorough washes. DAPI (Sigma-Aldrich, Cat No: D9542) was applied after further thorough washes. Antibody conditions are described in Table 1. Fibronectin was also stained in BMDMs using rabbit anti-mouse antibody (Abcam, Cat No: ab2413) followed by Alexa Fluor® 488 AffiniPure Donkey Anti-Rabbit IgG (H+L) (Jackson ImmunoResearch, Cambridge, UK, Cat No: 712-545-150). Incubation with secondary antibody alone was used as negative control.

Total cells were counted using DAPI^+^ cells, and myofibroblasts were defined as α-SMA^+^ cells with a fibroblastic cell shape (elongated, stress fibres present). Each treatment group was performed in triplicate, in each independent experiment. Three images per well were taken at random. The data was confirmed by an independent, blinded researcher.

### Western blot

BMDMs were re-suspended in radioimmunoprecipitation assay (RIPA) buffer supplemented with 1% proteinase inhibitor cocktail (PIC; Sigma-Aldrich, Cat No: P8340). Protein concentration was determined using Pierce BCA assay kit (Thermo Fisher Scientific, Cat No: 23225). Protein expression of α-SMA (host: rabbit, dilution: 1:1000, Abcam, Cat No: ab5694) was investigated, using goat anti-rabbit IgG H&L (HRP) at a dilution of 1:7000 (Abcam, Cat No: ab6721) as a secondary antibody. Housekeeping protein Rab11 (dilution: 1:250 Abcam, Cat No: ab151279) was used as a loading control (secondary antibody—goat anti-rabbit IgG as detailed above).

### Data analysis

Graph Pad Prism (V6, GraphPad Software, San Diego, USA) was used to create graphs and conduct statistical analysis. Data was tested for normality, and variances were tested to ensure similarity. This was conducted by Shapiro-Wilks test and Bartlett’s test. Analysis of statistical significance between two groups was conducted via an independent Student’s *t* test. one-way or two-way ANOVA was used where appropriate. Bonferroni correction was used for multiple comparison testing.

## Results

1. F4/80^+^ macrophages express α-smooth muscle actin (α-SMA) in the subretinal fibrotic lesion

**Fig. 1:**
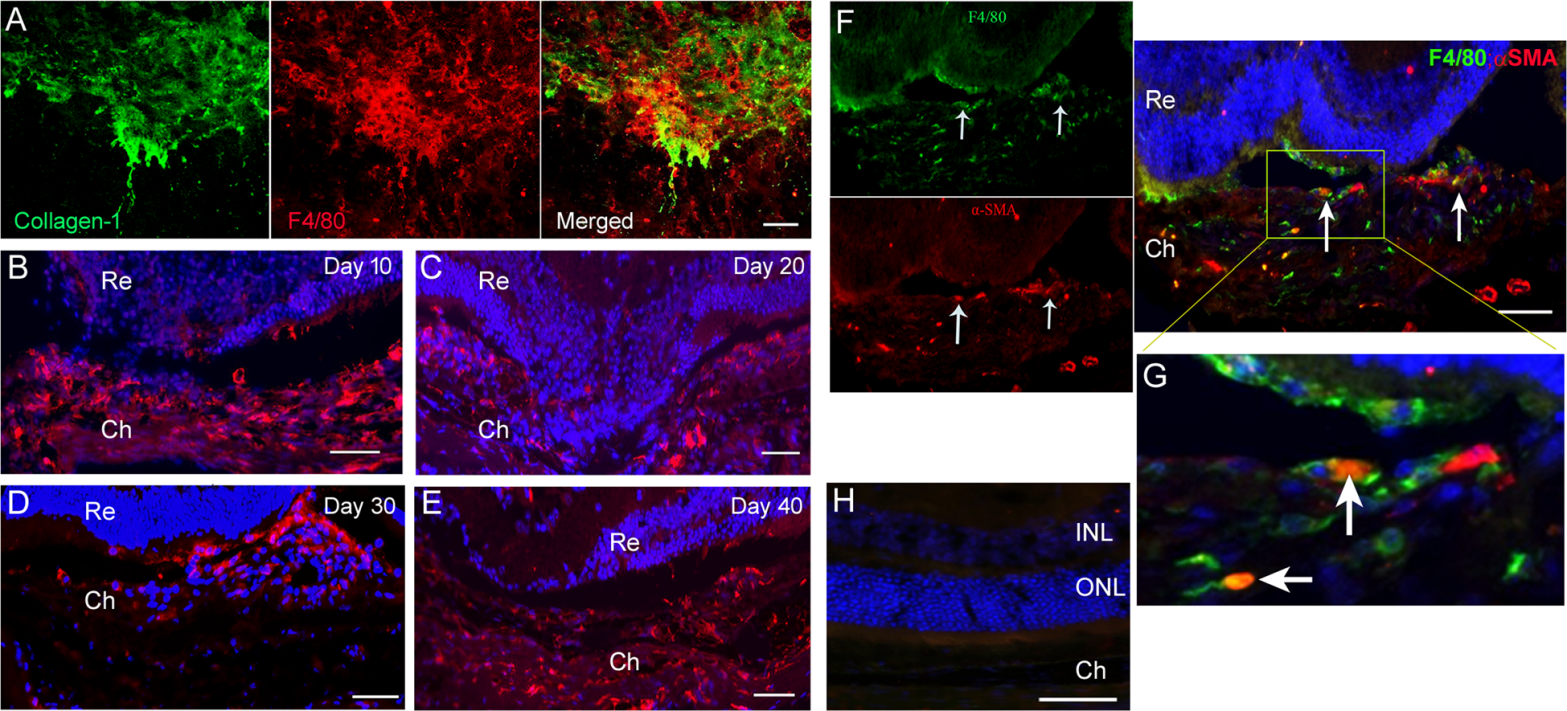
α-SMA^+^ macrophages in subretinal fibrotic lesions. **a** RPE/choroid flatmounts from 30 days post the second laser-treated eyes were stained for collagen-1 and F4/80 imaged by confocal microscopy. **b**–**e** Wax-embedded mouse eyes from day 10 (**b**), 20 (**c**), 30 (**d**) and 40 (**e**) of the two-stage laser fibrosis model were stained for F4/80 cells and DAPI and imaged by confocal microscopy. **f** Wax-embedded sections from 30 days post the two-stage laser model were stained for F4/80 (green) and α-SMA (red) and imaged by confocal microscopy. Arrows indicating F4/80^+^α-SMA^+^ cells. Scale bar = 100 μm. **g** Zoomed in area shown in yellow box in **f**. **h** Negative control staining. Ch—choroid; INL—inner nuclear layer; ONL—outer nuclear layer; Re—retina

In the two-stage laser model of subretinal fibrosis, infiltrating F4/80^+^ macrophages were detected at the lesion site throughout the disease course (Fig. 1). The infiltrating cells were present on the lesion surface as demonstrated in RPE/choroidal/sclera flatmount (Fig. 1a) as well as inside the lesion (Fig. 1b–e). Interestingly, we found that approximately 30% of F4/80^+^ cells co-express α-SMA (arrows in Fig. 1f and high-magnification images in Fig. 1g). The co-localisation of F4/80 and α-SMA was further confirmed using the Pixel Intensity Spatial Correlation Analysis in ImageJ (Pearson’s coefficient = 0.74, *n* = 3, Additional file 1 Figure S1A). Around the lesion area, some pigmented cells (likely RPE cells) were also α-SMA^+^ (Additional file 2: Figure S2). Our results suggest the heterogenic origin of myofibroblasts in subretinal fibrosis and may include MMT and EMT.

2. F4/80^+^α-SMA^+^ cells were detected in human macular fibrosis lesion

**Fig. 2:**
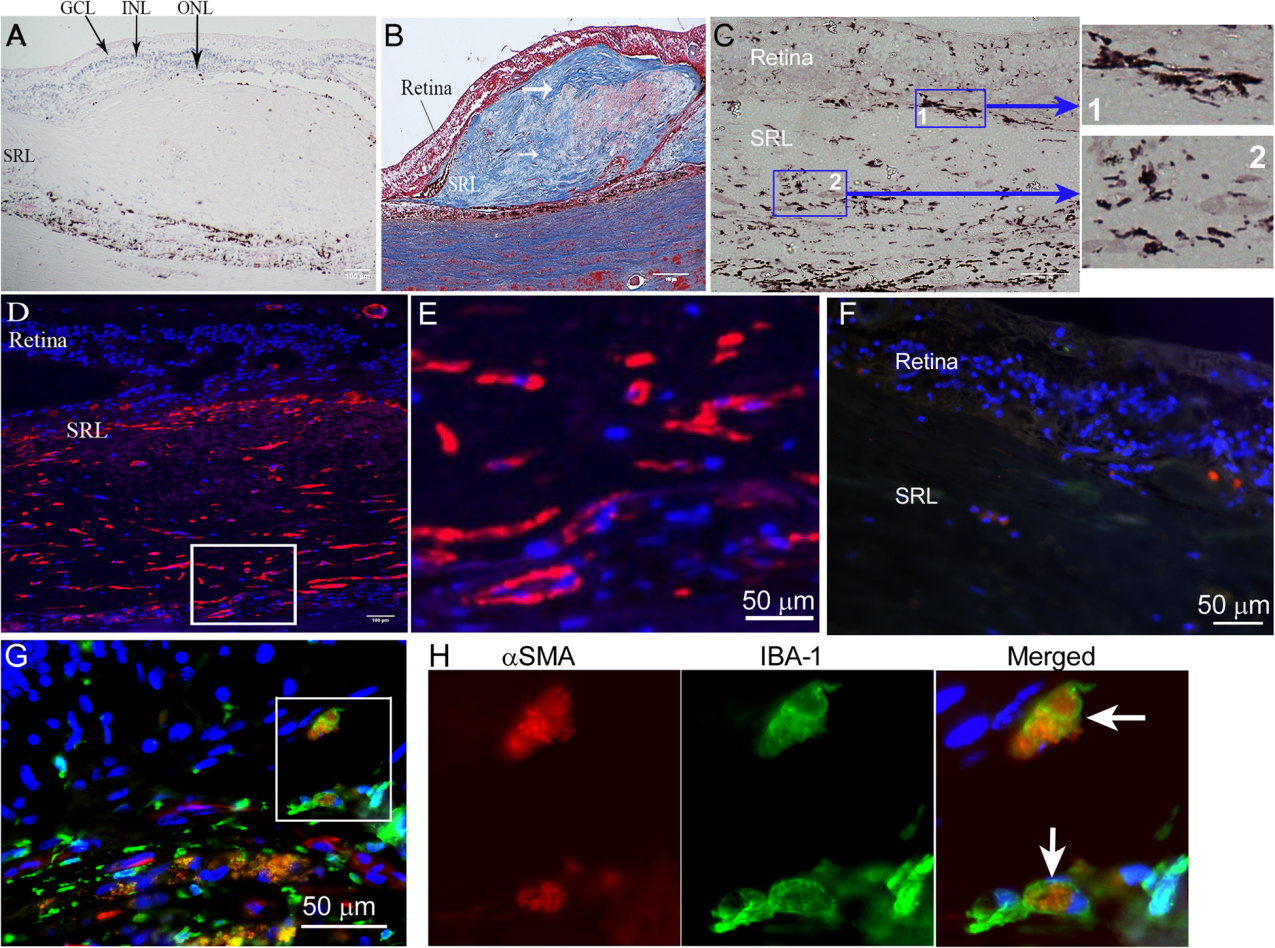
α-SMA^+^ and Iba-1^+^ cells in human macular fibrosis. **a** Haematoxylin and eosin staining showing subretinal lesion in a nAMD patient. **b** Masson’s trichrome staining showing subretinal fibrotic lesion. White arrows indicating collagen (blue)-rich lesions. **c** Retinal sections from nAMD patients were stained for Iba-1 (brown) and imaged by light microscopy. Blue box-1 showing Iba-1^+^ cells in retinal boundary to subretinal lesion. Box-2 showing Iba-1^+^ cells inside the fibrotic lesion. **d** Human subretinal lesion stained for α-SMA (red). **e** Higher magnification image of the area depicted by the white box in (**d**) showing α-SMA^+^ cells in the subretinal lesion. Scale bars = 100 um in a-d. **f** Negative control staining for Iba-1 and α-SMA in a human subretinal fibrotic lesion. **g** Confocal image of human subretinal fibrotic lesion stained for α-SMA (red) and Iba-1 (green). **h** Magnified area of box in **g** showing α-SMA^+^ Iba-1^+^ ‘MMT’ cells. GCL—ganglion cell layer; INL—inner nuclear layer; ONL—outer nuclear layer; SRL—subretinal lesion

To understand if the phenomenon of MMT also exists in human macular fibrosis, we conducted dual staining of Iba-1 and α-SMA in nAMD eyes. H+E and trichrome staining were used to identify subretinal lesion (SRL; Fig. 2a, b). Trichrome staining showed a well-contained lesion with a large amount of collagen fibres (blue), blood vessels and infiltrating cells (Fig. 2b). The infiltrating Iba-1^+^ cells were detected in the junction between the retina and SRL (blue box 1 with high-magnification image, Fig. 2c) as well as inside the lesion (blue box 2 with high-magnification image, Fig. 2c). Immunofluorescence uncovered multiple α-SMA^+^ cells in SRL (Fig. 2d with high magnification of boxed area in Fig. 2e). Dual staining for α-SMA (red) and Iba-1^+^ (green) identified several dual positive cells (Fig. 2g, boxed area with high magnification in Fig. 2h, negative control staining in Fig. 2f), making up approximately 20% of infiltrating Iba-1^+^ cells at the lesion. This data suggests that MMT is present in macular fibrosis secondary to nAMD.

3. C3a but not C5a induces MMT in bone marrow-derived macrophages

Previously, we have found that higher plasma levels of C3a and C5a are related to macular fibrosis in nAMD [24] and C3a is known to be able to induce EMT [26]. We were then interested to know if C3a or C5a contributes to MMT. Flow cytometry confirmed that the BMDMs were double positive for CD11b and F4/80 (data not shown). BMDMs were treated with C3a or C5a for 48–96 h. TGF-β1 (10 ng/ml) was used as a positive control. Phase-contrast microscopic investigation uncovered several elongated stretched cells in the TGF-β1- and C3a-treated groups (Additional file 3: Figure S3A, arrows) but not in the C5a-treated group (data not shown). qPCR showed a significant upregulation of α-SMA mRNA in TGF-β- and C3a-treated cells but not in C5a-treated cells (Additional Figure 3B). This was further confirmed by counting α-SMA^+^ cells to identify the percentage of cells which were differentiated myofibroblasts (Additional file 3: Figure S3C, D). Therefore, in the rest of our study, we focused on the effect of C3a-induced MMT in the context of retinal fibrosis.

**Fig. 3:**
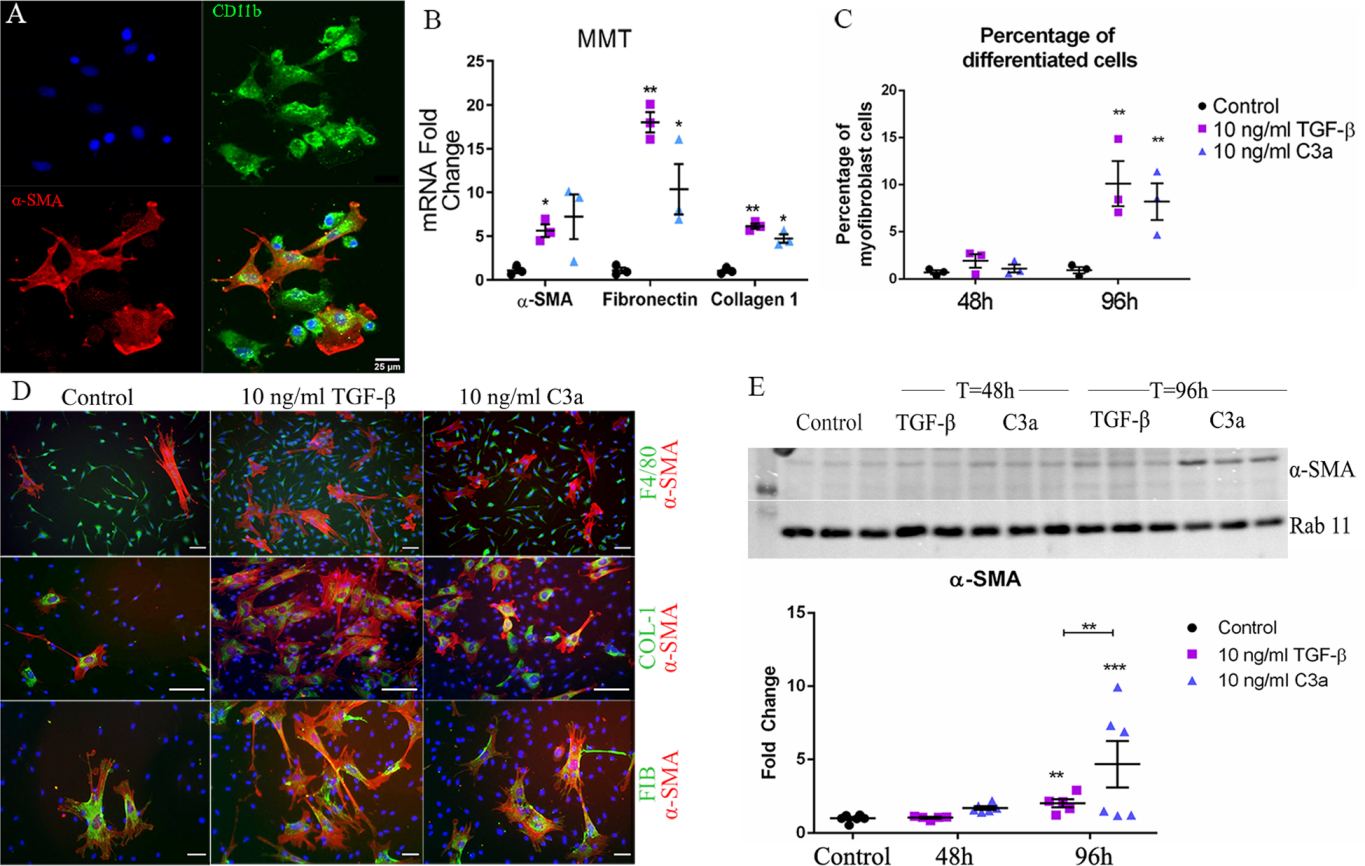
The effects of Complement C3a on BMDMs. BMDMs were treated with 10 ng/ml recombinant TGF-β1 or C3a, and the expression of various myofibroblast markers was investigated. **a** A confocal image showing CD11b^+^α-SMA^+^ cells (arrows) in BMDM cultures 48 h after C3a treatment. Scal bar = 25 um. **b** qPCR analysis of myofibroblast gene expression after 96 h of TGF-β or C3a treatment. Data presented as mean ± SEM, *n* = 3 samples, representative of 2 independent experiments. Statistical analysis = Student’s *t* test—*p < 0.05 treated vs control, **p < 0.01 treated vs control. **c** The percentage of α-SMA^+^ cells in BMDM culture after TGF-β or C3a treatment. Data presented as mean ± SEM. *n* = 3, data shown is representative of 2 independent experiments. **p < 0.01 compared to untreated control of the same time point, Student’s *t* test. **d** Representative confocal images of BMDM cultures treated with recombinant TGF-β1 or recombinant C3a for 96 h stained for α-SMA and F4/80 (upper panel) or collagen-1 and α-SMA (middle panel) or fibronectin and α-SMA (lower panel). Scale bars = 50 μm. **e** Western blot analysis of α-SMA after 48- or 96-h treatment with TGF-β1 or C3a. Image has been cropped for clarity. Data presented as fold change in α-SMA expression compared to housekeeping protein (rab11), mean ± SEM, *n* = 5–6 samples from 2 independent experiments, **p < 0.01, ***p < 0.005 compared to control untreated cells, Student’s *t* test

To further confirm C3a-induced MMT in BMDM cultures, we conducted dual staining of CD11b and α-SMA in BMDMs treated with C3a for 48 h. CD11b^+^α-SMA^+^ cells were identified by confocal microscopy (Fig. 3a), and the co-localisation was confirmed using the pixel intensity spatial correlation analysis in ImageJ (Pearson’s coefficient = 0.61, *n* = 6, Additional file 1: Figure S1B).

In addition to α-SMA, we also examined the expression of fibronectin and collagen-1 in C3a- or TGF-β1-treated cells. qPCR showed a significant upregulation of fibronectin and collagen-1 (as well as α-SMA) in TGF-β1- or C3a-treated cells although the effect of TGF-β1 appeared to be stronger than C3a (Fig. 3b). Immunohistochemistry showed that between 7–15% and 5–10% of F4/80^+^ BMDMs were differentiated to α-SMA^+^ myofibroblasts 96 h after TGF-β1 and C3a treatment, respectively, (Fig. 3c, d). All α-SMA^+^ cells were positive for fibronectin and collagen-1, although collagen-1^+^ α-SMA^-^ cells were occasionally observed (arrows, Fig. 3d). The TGF-β1- and C3a-mediated upregulation of α-SMA at 96 h was further confirmed by Western Blot (Fig. 3e). A significantly larger increase of α-SMA was seen after 96 h treatment with C3a, than after treatment with TGF-β1 (Fig. 3e). Representative raw blotting images for α-SMA and Rab11 are shown in Additional file 5: Figures S5 and Additional file 6: Figure S6, respectively).

Together, these data suggest that TGF-β and complement C3a can induce MMT.

4. C3a induced MMT is mediated through the C3a-C3aR signalling pathway

**Fig. 4:**
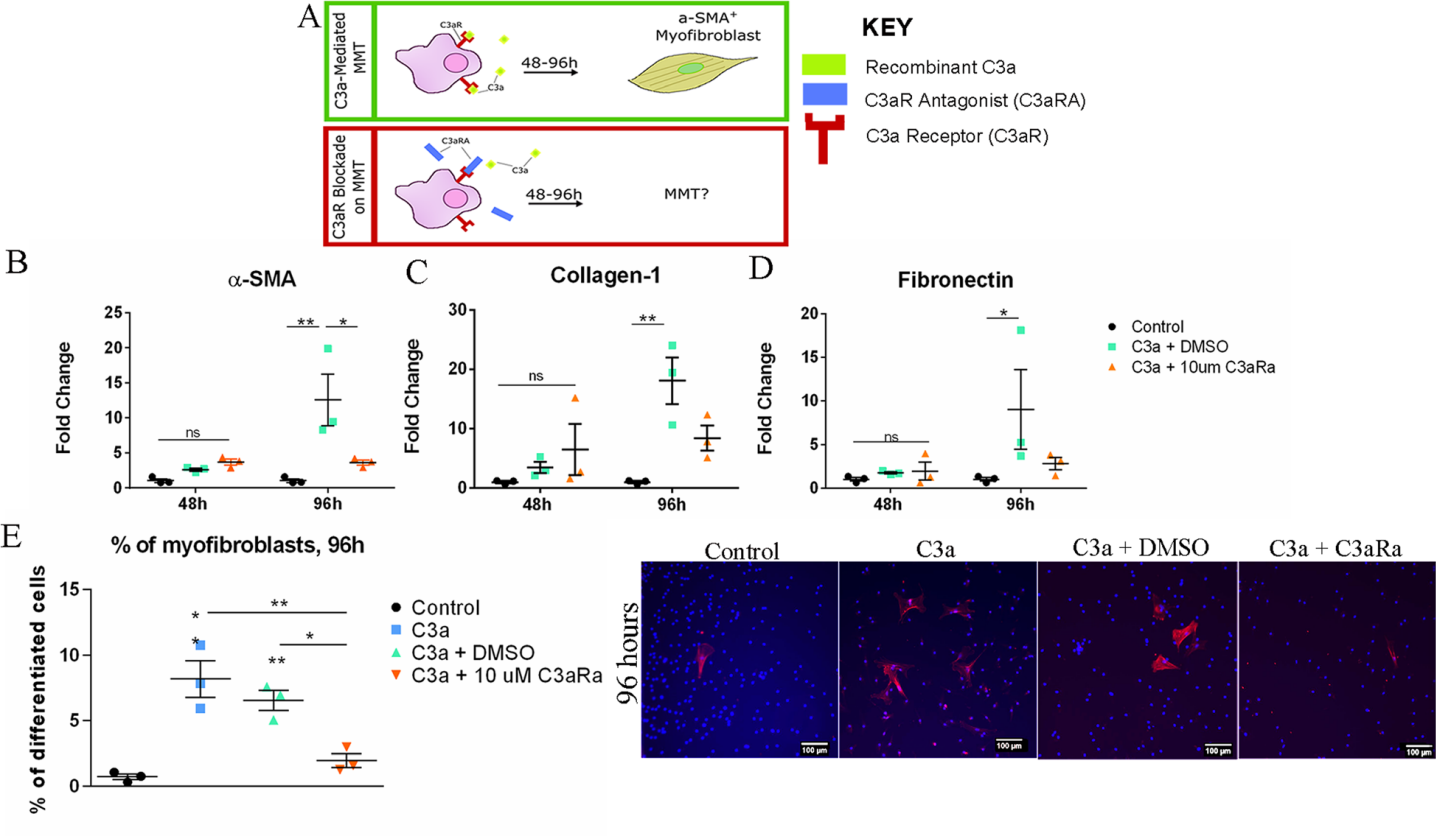
The effect of C3aRA on C3a-induced MMT. **a** Schematic showing that macrophages treated with complement C3a for 96 h undergo differentiation to a-SMA^+^ myofibroblasts. In the present study, we investigated the effect of adding both recombinant C3a and a C3aR antagonist (C3aRA). **b**–**d** Total RNA was extracted from BMDMs 48 and 96 h after treatment with C3a (10 ng/ml) plus C3aRA (10uM) or vehicle (DMSO). Control group consisted of untreated BMDMs. The expression of α-SMA (**b**), collagen-1 (**c**) and fibrolectin (**d**) mRNA was investigated by qPCR. Mean ± SEM. *p < 0.05, **p < 0.01. Two-way ANOVA, Bonferroni corrected. **e** Percentage of α-SMA^+^ cells in BMDM cultures following 96 h of C3a, C3a + DMSO and C3a + C3aRa treatments. *n* = 3, representative of the two independent experiments. Mean ± SEM. One-way ANOVA with Bonferroni correction, The asterisk is indicated above the group, significance is compared to the control group (untreated BMDMs). Where the asterisk is indicated between two conditions, significance stated is that between the two groups. *p < 0.05, **p < 0.01. Representative images of α-SMA^+^ cells (red) in different groups are shown in the left panel.

Macrophages are known to express C3aR [31]. To understand if C3a-induced MMT is mediated through the C3aR signalling pathway, a C3aR-specific antagonist (C3aRA) was used in our study (Fig. 4a).

Similar to previous observations, C3a-induced upregulation of α-SMA, collagen-1 and fibronectin was observed at 96 h (Fig. 4b–d). Treatment with C3aRA suppressed C3a-mediated upregulation of α-SMA (Fig. 4b), collagen-1 (Fig. 4c) and fibronectin (Fig. 4d). The reduced expression of a-SMA by C3aRA in C3a-treated BMDMs was further confirmed by immunocytochemistry (Fig. 4e).

5. TGF-β, complement C3, C3a and C3aR are upregulated in the subretinal fibrotic lesion

To understand the molecular pathways involved in MMT in subretinal fibrosis, we examined TGF-β, C3a and C3aR expression in subretinal lesions of the two-stage laser model [9]. TGF-β mRNA was significantly increased in the retina 3 and 25 days post the second laser treatment (Additional file 4: Figure S4A). In addition, TGF-β mRNA was significantly upregulated in the RPE/choroid/sclera layer 25 days post the second laser treatment (Additional file 4 Figure S4B). Confocal microscopy detected TGF-β in normal mouse retina, including RPE cells (Additional file 4 Figure S4C). Increased TGF-β staining was seen in the retina and subretinal lesion 30 days after the second laser treatment (Additional Figure 4D, white arrow).

**Fig. 5:**
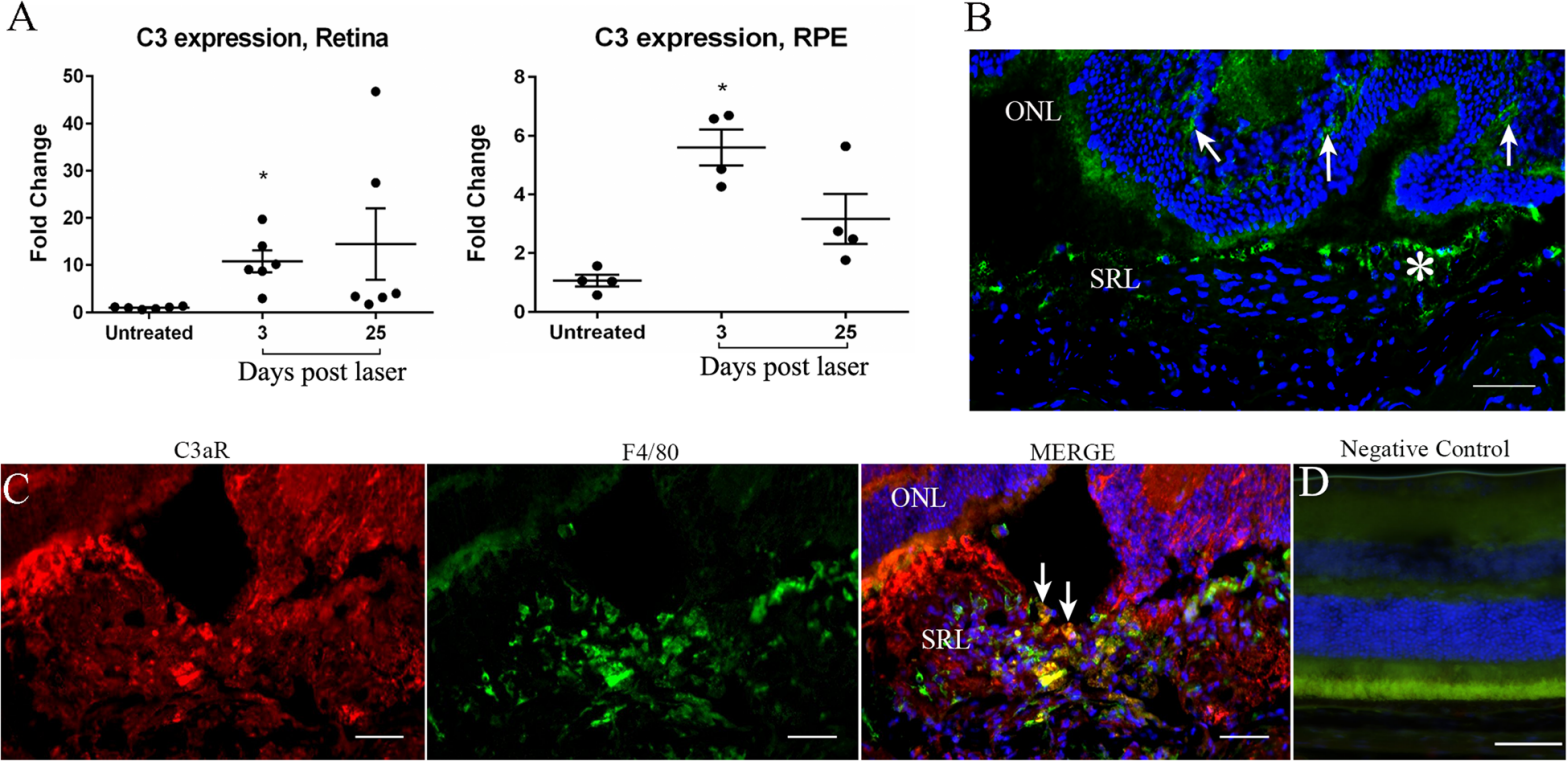
Complement C3/ C3a expression in subretinal fibrosis. **a** The expression of C3 mRNA in the retina and RPE/choroid/sclera was examined by qPCR at 3- and 25-days post the second laser. Mean ± SEM. *n* = 4–6 eyes per group. *p < 0.05 compared to untreated controls. One-way ANOVA, Bonferroni corrected. **b**, **c** Cryosections from a mouse eye 30 days post two-stage laser model were stained for C3a (**b**), C3aR (red) and F4/80 (green) (**c**) and imaged by confocal microscopy. **b** C3a staining in the retina (arrows) and in the subretinal lesion (asterisk). **c** F4/80^+^C3aR^+^ macrophages within the subretinal lesion (yellow cells, arrows). ONL = outer nuclear layer, SRL = subretinal lesion. Scale bars = 100 μm.

We observed a significant increase in C3 gene expression in the RPE/Choroid/sclera 3 days after the second laser treatment, compared to tissues from matched non-lasered control mice (Fig. 5a). Thirty days post the second laser treatment, complement C3a is present in the retina (Fig. 5b, arrows) and within the subretinal lesion (Fig. 5b, yellow arrow). C3aR was also detected in the lesion (Fig. 5c) and some of the C3aR^+^ cells were F4/80^+^ macrophages (Fig. 5c). Negative control staining is showed in Fig. 5d. Our results suggest that TGF-β, C3a and C3aR are present within the lesion in this model of subretinal fibrosis.

6. The effect of C3a blockade on subretinal fibrosis

**Fig. 6:**
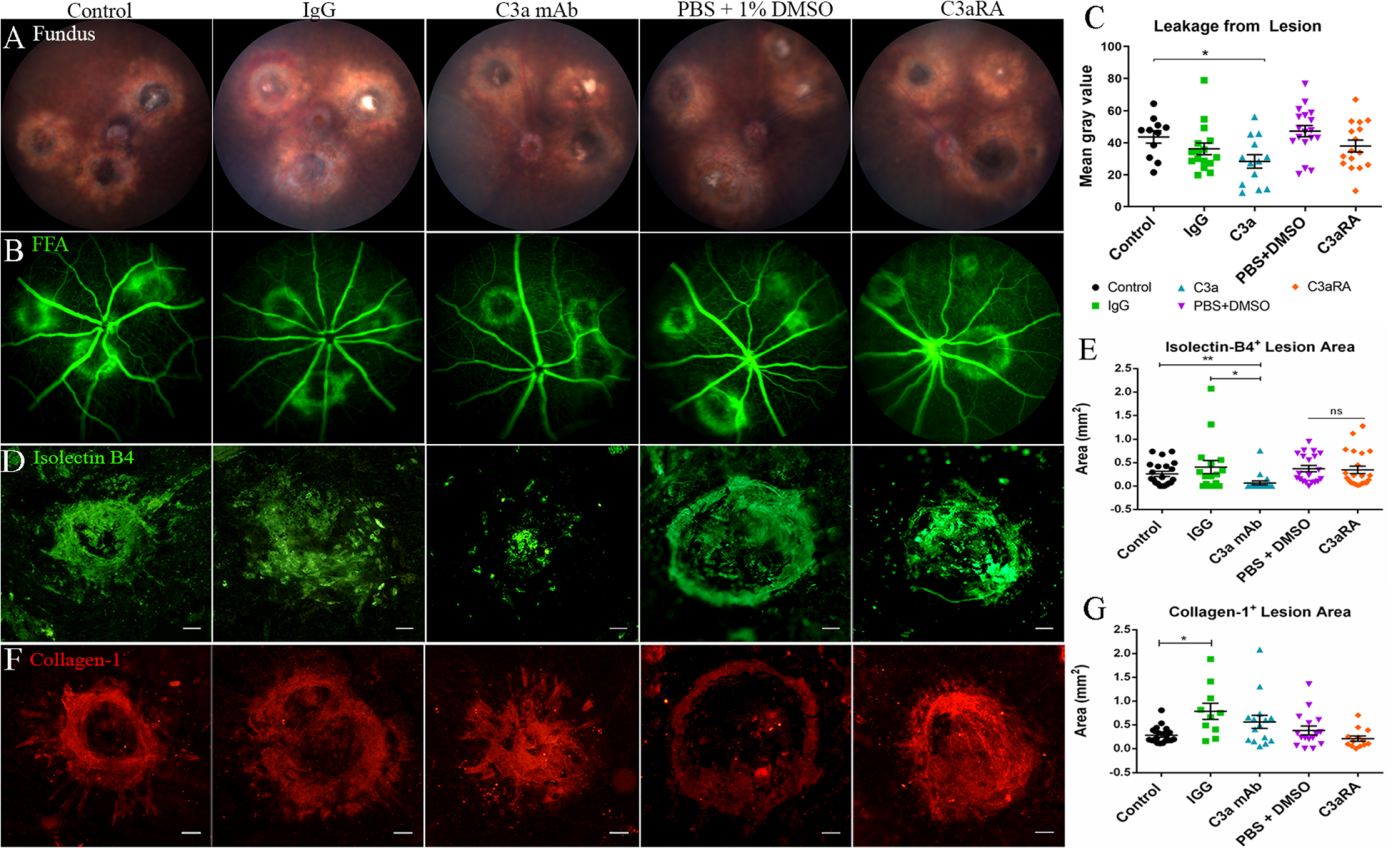
The effect of C3a blockade on a mouse model of subretinal fibrosis. Representative fundus (**a**) and FFA (**b**) images at 20 days post the two-stage laser subretinal fibrosis model from mice that were untreated (control) or treated with rat IgG, or anti-C3a mAb or C3aRA or vehicle (DMSO). **c** Quantitative measurement of fluorescein leakage at 20 days p.l. expressed as mean of gray value. Mean ± SEM, *n* = 6 eyes per group, 11–18 lesions per group. One-way ANOVA, Bonferroni corrected, *p < 0.05, vs untreated controls. **d**–**g** RPE/chroid flatmounts from 30 days two-stage laser model treated with C3a blocking agents were stained for isolectin B4 (**d**) or collagen-1 (**f**) and imaged by confocal microscopy. Scale bar = 100 μm. **e** Quantitative measurement of isolectin B4^+^ lesion area in different groups. **g** Quantitative measurement of collagen-1^+^ lesion area in different groups. Mean ± SEM. Collagen-1: *n* = 10–20 lesions per group, 3–5 eyes. Isolectin-B4: *n* = 16–22 lesions per group, 5–7 eyes. *p < 0.05. **p < 0.01. ns = non-significant.one-way ANOVA, Bonferroni corrected

To further understand the contribution of the C3a/C3aR pathway in retinal fibrosis, we used C3a mAb and a C3aR antagonist (C3aRA) in our two-stage laser mouse model of subretinal fibrosis [9]. Clinical examination showed that fluorescence leakage from subretinal fibrovascular lesions was significantly reduced 20 days after C3a mAb treatment (Fig. 6a–c). C3aRA treatment did not significantly reduce fluorescence leakage compared to the vehicle (DMSO) group (Fig. 6a–c).

Subretinal fibrovascular lesions were further examined in RPE/choroid/sclera flatmounts 30 days after the second laser treatment (10 days after the second intravitreal treatment) following Isolectin-B4 (Fig. 6d, e) and Collagen-1 (Fig. 6f, g) staining. A significant reduction in Isolectin-B4^+^ area was seen between IgG and C3a mAb groups (0.40 ± 0.14 mm^2^ vs 0.06 ± 0.04 mm^2^) (Fig. 6e). There was no significant difference between the vehicle (DMSO) group and C3aRA group (0.35 ± 0.07 mm^2^ vs 0.29 ± 0.08 mm^2^) (Fig. 6e). This data showed that blocking C3a with a monoclonal antibody significantly reduces the vascular component of the subretinal lesion 30 days post laser treatment.

Intravitreal injection of IgG significantly increased collagen-1^+^ lesions compared with non-treatment controls (Fig. 6f, g). Compared to the IgG group, anti-C3a mAb treatment insignificantly reduced collagen-1^+^ area (Fig. 6g) (IgG 0.78 ± 0.17 mm^2^ vs C3a mAb 0.48 ± 0.14 mm^2^). There was no difference in collagen-1 lesion size between vehicle (DMSO) and C3aRA treated eyes (0.37 ± 0.09 mm^2^ vs 0.31 ± 0.05 mm^2^) (Fig. 6f, g).

## Discussion

Neovasculature in nAMD often progresses into fibrovascular lesions (macular fibrosis) even after anti-VEGF therapy [32]. Inflammation, in particular the innate immune responses including macrophage infiltration and complement activation, is critically involved in the development and progression of nAMD [21, 23, 33]. In this study, we show that infiltrating macrophages may participate in fibrovascular lesion development through transdifferentiating into myofibroblasts (i.e. MMT) and TGF-β and complement C3a may contribute to this process.

A persistent low-grade inflammation in the vascular lesion site is believed to be a key driver of macular fibrosis [8]. The inflammatory response can not only damage macular cells (e.g., RPE and photoreceptors), but also lead to the release of various soluble mediators, including proinflammatory and profibrotic factors. These mediators can initiate a cascade within the cellular milieu that leads to the accumulation of ECM, rich in fibrillary collagens and fibronectin in the deposition of diseased blood vessels. Macrophages are known to actively participate in tissue fibrosis [34]. In the disease initiation stage, the classically activated macrophages promote inflammation and tissue damage, whereas in the disease resolving stage, alternatively activated macrophages suppress inflammation, promote tissue repair and wound healing [35]. Dysregulated macrophage function, either in the disease initiation or in the resolving stage may lead to persistent inflammation, pathogenic wound healing and fibrosis. Macrophages may also promote fibrosis through their transition to myofibroblasts. This phenomenon was previously observed in kidney fibrosis [17–19]. A key marker of MMT is the co-expression of macrophage marker F4/80 and myofibroblast marker α-SMA. We found around 20% of Iba-1^+^ macrophages co-express α-SMA in human nAMD eyes and 30% (of F4/80^+^ macrophages) in our mouse model of subretinal fibrosis. Our results suggest that infiltrating macrophages may participate in macular fibrosis by converting themselves into myofibroblasts.

Besides macrophages, other cells may give rise to myofibroblasts in macular fibrosis. RPE cells have previously been shown to undergo EMT [36], and we observed α-SMA^+^ RPE-like cells in the mouse model of subretinal fibrosis (Additional file 2 Figure S2), suggesting that RPE may contribute to macular fibrosis through EMT. Myofibroblasts may also originate from endothelial cells through endothelial to mesenchymal transition (EndoMT) [8]. Other cells of interest include glial cells, circulating fibrocytes and choroidal stromal cells [8]. Further research is required to fully understand the origin of α-SMA^+^ myofibroblasts in macular fibrosis and the molecular pathways involved in their recruitment and activation.

TGF-β is a well-established profibrotic mediator that can induce mesenchymal transition in a variety of cells including epithelial cells [37], endothelial cells [38] and macrophages [19]. RPE cells are known to constitutively express TGF-β to maintain the immune suppressive microenvironment of the subretinal space [39]. Recently, using single-cell RNA sequencing analysis, we reported that TGF-β1 and TGF-β2 mRNA was expressed predominately by retinal microglia and Müller cells, respectively [40]. In the current study, we found that the expression of TGF-β1 in the retina and RPE was increased 3 and 25 days after the second laser treatment in our model of subretinal fibrosis. TGF-β may be a key driver of MMT in retinal fibrosis.

Another interesting observation of this study is that complement C3a but not C5a induced MMT. Approximately 5–10% of C3a-treated macrophages expressed α-SMA, fibronectin and collagen-1, and this C3a-mediated MMT can be prevented by C3aR antagonist C3aRA. C3a has been reported to induce mesenchymal transition in PTECs [26]. A previous study from our group has reported higher plasma levels of C3a, C4a and C5a in nAMD patients with macular fibrosis [24]. These data suggest that C3a may participate in macular fibrosis in nAMD through induction of MMT. However, in the mouse model of subretinal fibrosis, blocking of C3a only suppressed the vascular component of subretinal lesion, but did not significantly reduce subretinal fibrosis. C3aRA also failed to suppress subretinal fibrosis. A previous study has shown that blocking C3a reduced CNV lesion size [25] and that C3a treatment increased VEGF production by RPE cells [25]. In addition, we previously observed higher plasma levels of C3a in nAMD patients who were partially responsive to anti-VEGF therapy compared with the full responders [24]. It is possible that the C3a-C3aR pathway may play a more important role in neovascularisation than in subretinal fibrosis. It is also possible that the lack of therapeutic effect is due to limitations of the treatment regime employed in our study. In this study, the intravitreal injections of C3a blockage were conducted at day 0 and day 20 post the second laser. In our experience, multiple intravitreal injection in mouse eyes with an interval time shorter than 2 weeks causes ocular trauma and inflammation that can be severe enough to affect retinal disease [27]. Indeed, the lesion size in our IgG treated group was significantly larger than that of intravitreal injection-free group (Fig. 6g). The 20-day interval between two injections, although minimized injection-related ocular trauma and inflammation, may result in insufficient intraocular neutralisation of C3a or C3aR. Further studies using controlled drug release device or long-lasting drugs will be needed to address this question.

## Conclusions

In conclusion, our study shows that MMT is involved in macular fibrosis secondary to nAMD. TGF-β and complement C3a (but not C5a) are potential inducers of MMT in macular fibrosis. Further studies on the importance of MMT in macular fibrosis, the key triggers of MMT, the macrophage subsets and molecular pathways involved in MMT will be essential to understand if MMT can be targeted for therapy.

## Supplementary Information

**Additional file 1: Figure S1.** Co-localisation analysis using Pearson’s coefficient. Pearson’s coefficient analysis for F4/80^+^α-SMA^+^ cells in the subretinal fibrosis model (*n* = 3 images). (B) Pearson’s coefficient analysis for CD11b^+^α-SMA^+^ cells in bone marrow-derived macrophages, 48 h after treatment with 10 ng/ml C3a (*n* = 6 images).

**Additional file 2: Figure S2.** Pigmented cells express α-SMA in subretinal fibrosis. Pigmented cells around the subretinal fibrotic lesion were positive for α-SMA (red).

**Additional file 3: Figure S3.** Complement C3a, but not C5a increased α-SMA expression in bone marrow-derived macrophages (BMDMs). BMDMs were treated with 10 ng/ml recombinant TGF-β1, C3a or C5a, and the expression of α-SMA was investigated. (A) After 96 h of treatment with TGF-β or C3a, large “stretched” cells are visible in the culture dish (arrows). Scale bar = 50 μm. (B) qPCR analysis of α-SMA gene expression after 96 h of TGF-β, C3a or C5a treatment. Mean ± SEM, *n* = 3 samples, representative of 2 independent experiments. Student’s *t* test, *p < 0.05 treated vs control, **p < 0.01 treated vs control. (C) Percentage of α-SMA^+^ cells in BMDM cultures 96 h after TGF-β or C3a treatment. Mean ± SEM. *n* = 3, data shown is representative of 2 independent experiments. **p < 0.01 compared with control untreated group. One-way ANOVA, Bonferroni corrected. (D) Representative images are shown to illustrate the data presented in (C) Scale bar = 100 μm.

**Additional file 4: Figure S4.** TGF-β expression in subretinal fibrosis. Expression of TGF-β in retina (A) and RPE/choroid tissue (B) was examined by qPCR at 3- and 25-days post the second laser. Mean ± SEM. *n* = 4–6 eyes per group. *p < 0.05 compared to untreated controls. One-way ANOVA, Bonferroni corrected. (C, D) Cryosections from a mouse eye 30 days post two-stage laser model were stained for TGF-β1 (red). In a normal area of the lasered eye, (C) a few retinal cells and RPE cells were detected positive for TGF-β1. (D) TGF-β immunoreactivities were observed in the subretinal lesion (white arrow). ONL = outer nuclear layer; RPE = retinal pigment epithelial layer; SRL = subretinal lesion. Scale bar = 100 μm

**Additional file 5: Figure S5.** Raw western blot Figure 3D α-SMA. Raw image of western blot data presented in figure 3D (α-SMA). Note that the image has been flipped in the main manuscript figure

**Additional file 6: Figure S6.** Raw western blot Figure 3D Rab11. Raw image of western blot data presented in figure 3D (Rab11). Note that the image has been flipped in the main manuscript figure

## Data Availability

Data sharing is not applicable to this article as no datasets were generated or analysed during the current study.

## Abbreviations

AMD: Age-related macular degeneration
MMT: Macrophage to myofibroblast transition
nAMD: Neovascular age-related macular degeneration
α-SMA: Alpha smooth muscle actin
VEGF: Vascular endothelial growth factor
TGF-β: Transforming growth factor beta
qPCR: Quantitative polymerase chain reaction
ECM: Extracellular matrix
CNV: Choroidal neovascularisation
EMT: Epithelial to mesenchymal transition
ARVO: Association for Research in Vision and Ophthalmology
RPE: Retinal pigment epithelium
TE: Tris-Edta
DAPI: 4′,6-Diamidino-2-phenylindole
FCS: Foetal calf serum
DMSO: Dimethyl sulfoxide
RNA: Ribonucleic acid
PFA: Paraformaldehyde
PBS: Phosphate-buffered saline
RIPA: Radioimmunoprecipitation assay
PIC: Proteinase inhibitor cocktail
HRP: Horseradish peroxidase
ANOVA: Analysis of Variance
