# Supplementary figures and images for: Macrophage to myofibroblast transition contributes to subretinal fibrosis secondary to neovascular age-related macular degeneration
Source: J Neuroinflammation. 2020 Nov 25;17:355. doi: 10.1186/s12974-020-02033-7 (PMC7690191; doi:10.1186/s12974-020-02033-7)

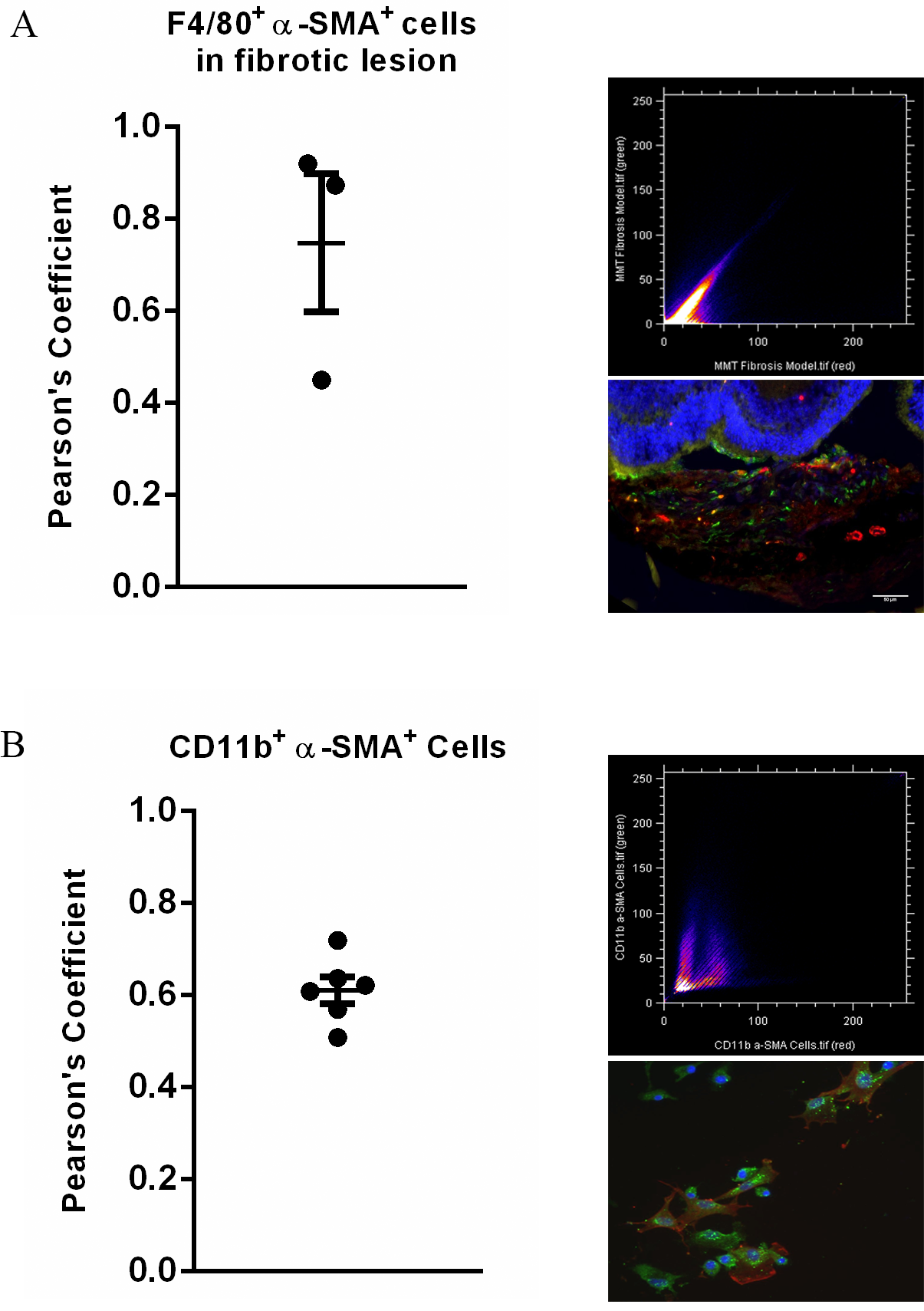

Supplement: Supplementary file 1 — Additional file 1: Figure S1. Co-localisation analysis using Pearson’s coefficient. Pearson’s coefficient analysis for F4/80+α-SMA+ cells in the subretinal fibrosis model (n = 3 images). (B) Pearson’s coefficient analysis for CD11b+α-SMA+ cells in bone marrow-derived macrophages, 48 h after treatment with 10 ng/ml C3a (n = 6 images). [file 12974_2020_2033_MOESM1_ESM.tif]

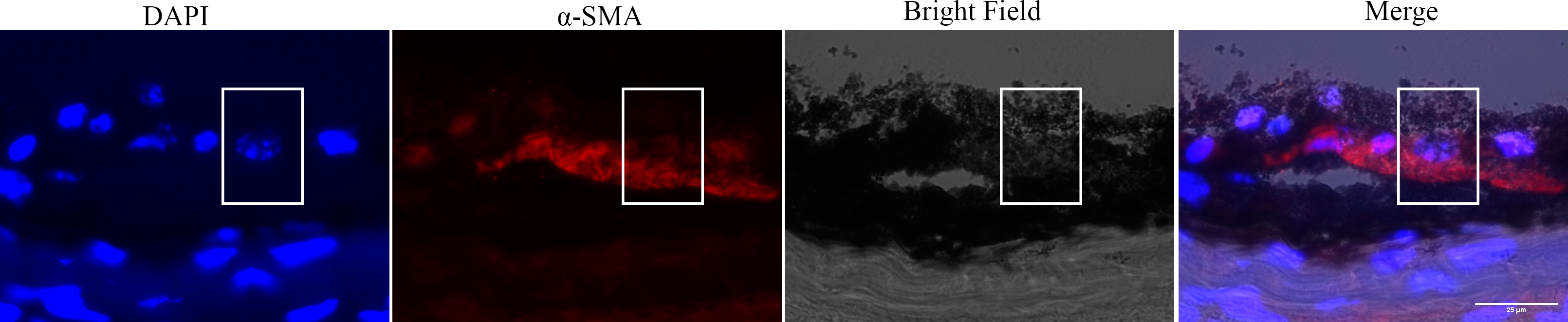

Supplement: Supplementary file 2 — Additional file 2: Figure S2. Pigmented cells express α-SMA in subretinal fibrosis. Pigmented cells around the subretinal fibrotic lesion were positive for α-SMA (red). [file 12974_2020_2033_MOESM2_ESM.tif]

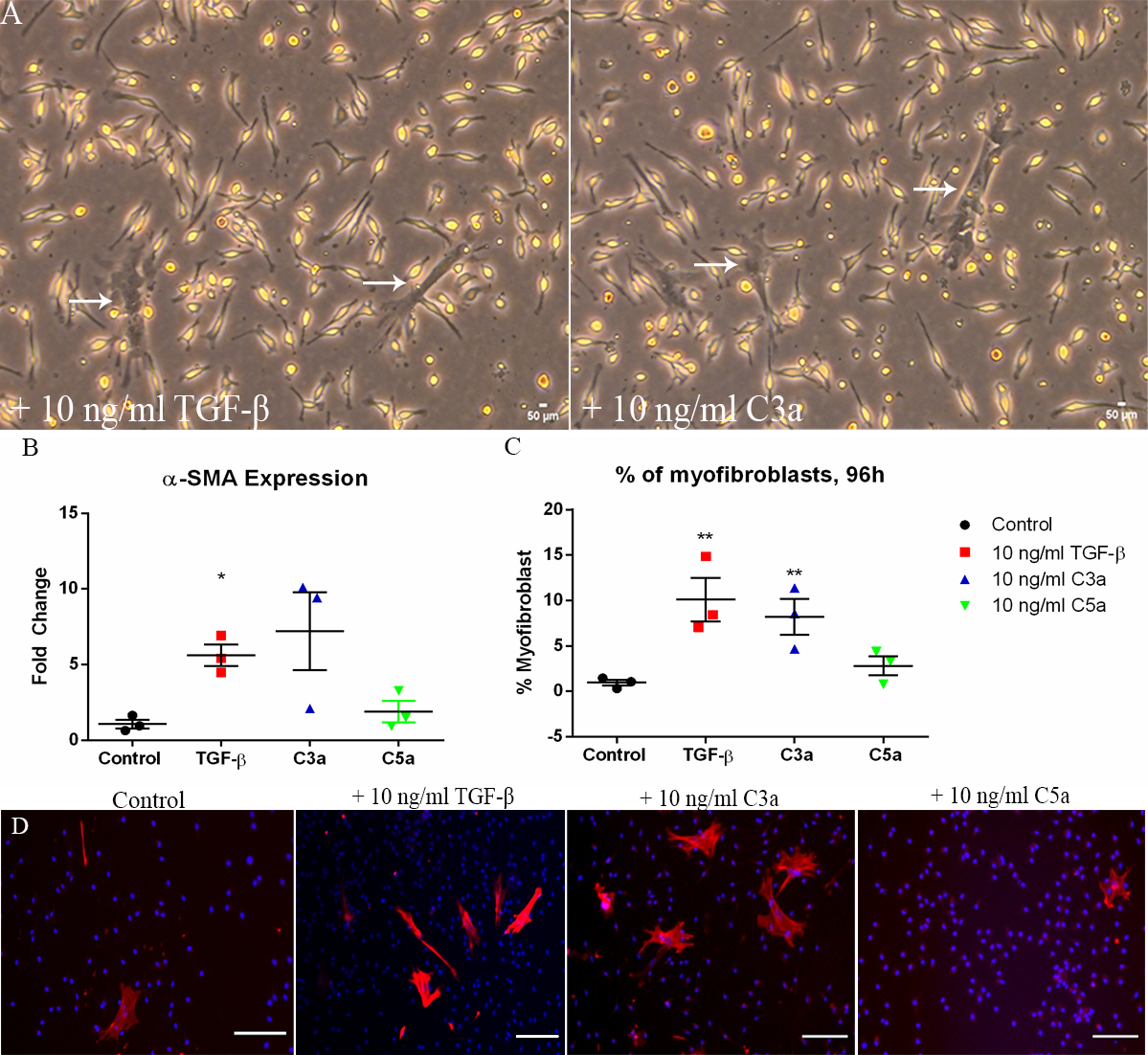

Supplement: Supplementary file 3 — Additional file 3: Figure S3. Complement C3a, but not C5a increased α-SMA expression in bone marrow-derived macrophages (BMDMs). BMDMs were treated with 10 ng/ml recombinant TGF-β1, C3a or C5a, and the expression of α-SMA was investigated. (A) After 96 h of treatment with TGF-β or C3a, large “stretched” cells are visible in the culture dish (arrows). Scale bar = 50 μm. (B) qPCR analysis of α-SMA gene expression after 96 h of TGF-β, C3a or C5a treatment. Mean ± SEM, n = 3 samples, representative of 2 independent experiments. Student’s t test, *p < 0.05 treated vs control, **p < 0.01 treated vs control. (C) Percentage of α-SMA+ cells in BMDM cultures 96 h after TGF-β or C3a treatment. Mean ± SEM. n = 3, data shown is representative of 2 independent experiments. **p < 0.01 compared with control untreated group. One-way ANOVA, Bonferroni corrected. (D) Representative images are shown to illustrate the data presented in (C) Scale bar = 100 μm. [file 12974_2020_2033_MOESM3_ESM.tif]

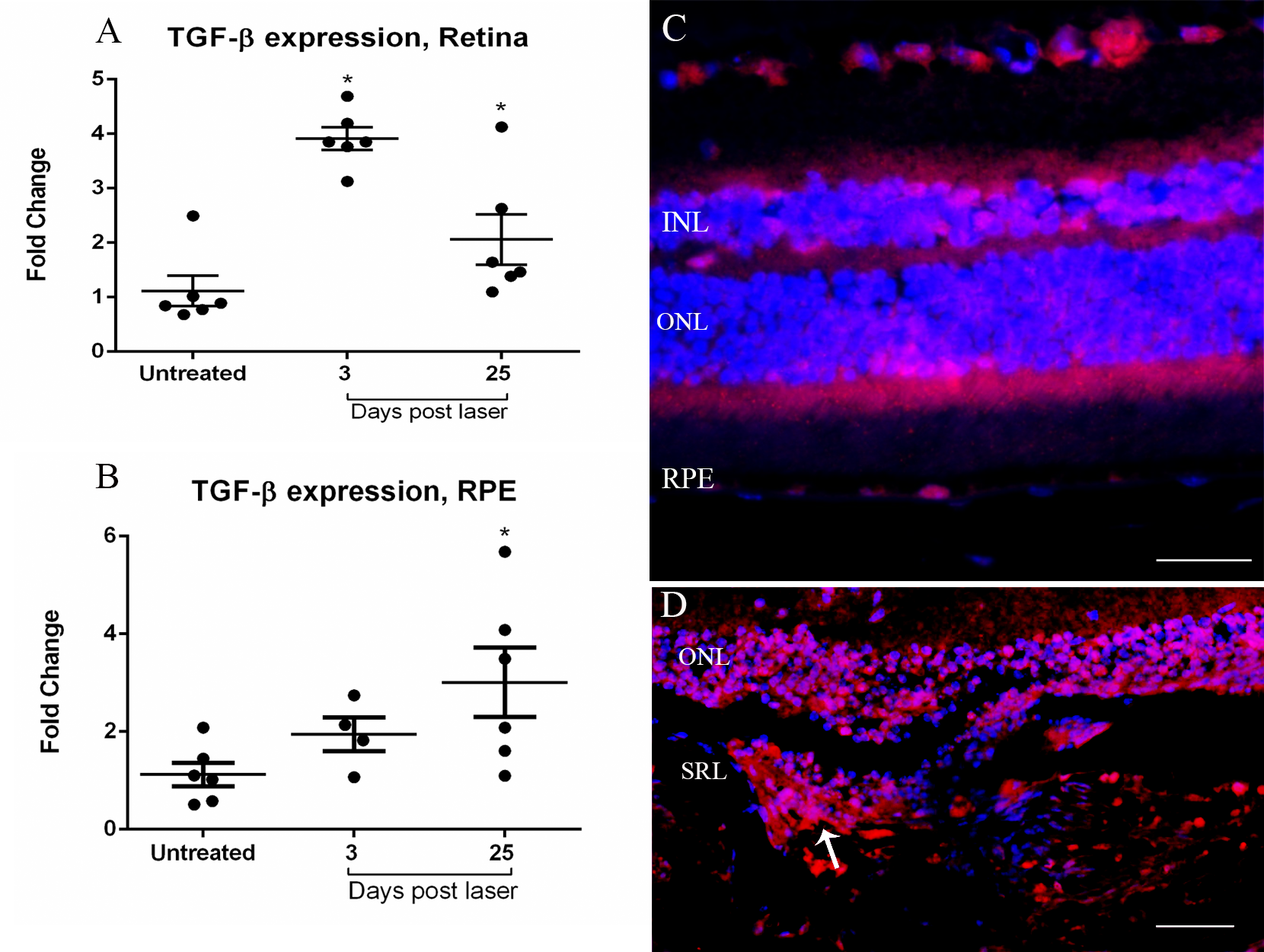

Supplement: Supplementary file 4 — Additional file 4: Figure S4. TGF-β expression in subretinal fibrosis. Expression of TGF-β in retina (A) and RPE/choroid tissue (B) was examined by qPCR at 3- and 25-days post the second laser. Mean ± SEM. n = 4–6 eyes per group. *p < 0.05 compared to untreated controls. One-way ANOVA, Bonferroni corrected. (C, D) Cryosections from a mouse eye 30 days post two-stage laser model were stained for TGF-β1 (red). In a normal area of the lasered eye, (C) a few retinal cells and RPE cells were detected positive for TGF-β1. (D) TGF-β immunoreactivities were observed in the subretinal lesion (white arrow). ONL = outer nuclear layer; RPE = retinal pigment epithelial layer; SRL = subretinal lesion. Scale bar = 100 μm [file 12974_2020_2033_MOESM4_ESM.tif]

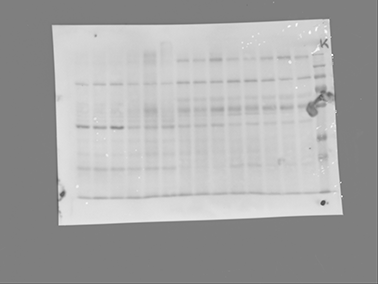

Supplement: Supplementary file 5 — Additional file 5: Figure S5. Raw western blot Figure 3D α-SMA. Raw image of western blot data presented in figure 3D (α-SMA). Note that the image has been flipped in the main manuscript figure [file 12974_2020_2033_MOESM5_ESM.tif]

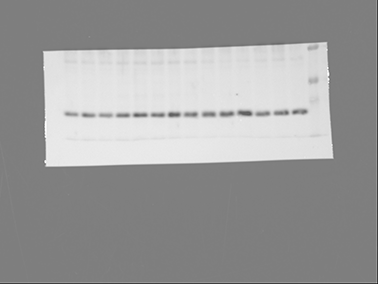

Supplement: Supplementary file 6 — Additional file 6: Figure S6. Raw western blot Figure 3D Rab11. Raw image of western blot data presented in figure 3D (Rab11). Note that the image has been flipped in the main manuscript figure [file 12974_2020_2033_MOESM6_ESM.tif]
